# Neurological Effects of Pesticide Use among Farmers in China

**DOI:** 10.3390/ijerph110403995

**Published:** 2014-04-14

**Authors:** Yifan Li, Chao Zhang, Yanhong Yin, Fang Cui, Jinyang Cai, Zhaohui Chen, Yanhong Jin, Mark G. Robson, Mao Li, Yuting Ren, Xusheng Huang, Ruifa Hu

**Affiliations:** 1 Department of Neurology, Chinese PLA General Hospital, No. 28 Fuxing Road, Haidian District, Beijing 100853, China; E-Mails: jessielee@sina.com (Y.L.); yanhongyin1013@gmail.com (Y.Y.); cuifang301@sina.com (F.C.); shentong100841@sina.com (Z.C.); limao301shennei@163.com (M.L.); rytlhx@126.com (Y.R.); 2 School of Management and Economics, Beijing Institute of Technology, 5 South Zhongguancun Street, Beijing 100081, China; E-Mails: zhangchao9501@163.com (C.Z.); caijinyang@bit.edu.cn (J.C.); 3 School of Environmental and Biological Sciences, Rutgers University, 59 Dudley Road, New Brunswick, NJ 08901, USA; E-Mails: yjin@sebs.rutgers.edu (Y.J.); robson@aesop.rutgers.edu (M.G.R.)

**Keywords:** China, health effect, neurological deficit, pesticide overuse

## Abstract

The intensive use of pesticides has attracted great attention from the Chinese government. However, current regulations have had limited influence on their safe use. Although the acute neurologic effects of pesticides have been well documented, little is known about their cumulative effects. Knowledge of the impact of pesticides on health may convince farmers to minimize their use. We conducted a cross-sectional study in three provinces of China to evaluate the relationship between pesticide exposure and neurological dysfunction. Crop farmers were divided into two groups depending on their level of pesticide exposure. A total of 236 participants were assessed by questionnaire and neurological examination for symptoms and signs of neuropathy. Characteristics of neurologic dysfunction following cumulative low-level exposure were assessed with logistic regression analysis. Farmers exposed to high-level pesticide use had greater risk of developing sensations of numbness or prickling (odds ratio (OR) 2.62, 95% confidence interval (CI): 1.08–6.36). After adjusting for recent exposure, the risk of numbness or prickling symptoms (OR 2.55, 95% CI: 1.04–6.25) remained statistically significant. Loss of muscle strength and decreased deep tendon reflexes had OR > 2, however, this did not reach statistical significance. These findings suggest that overuse of pesticides increased risk of neurologic dysfunction among farmers, with somatosensory small fibers most likely affected. Measures that are more efficient should be taken to curb excessive use of pesticides.

## 1. Introduction

China has been the global leader in pesticide use since the 1990s [1]. Use of chemical pesticides per hectare in China is almost three-fold greater than in developed countries [2,3,4]. This intensive use poses serious problems for the food chain and ecological environment due to the presence of pesticide residues [5,6]. Despite efforts made by the Chinese government to promote the safe use of pesticides, the amount of pesticide used in agricultural production has been rapidly rising in recent years [7]. In addition to disseminating rules and regulations associated with pesticide use, better understanding of the impact of pesticides on human health may encourage farmers reduce its use. To date, few studies have examined the impact of pesticide use on the health of farmers in China.

Neurologic dysfunction is one of the most prominent health effects of pesticide exposure. Organophosphate (OP), carbamate, and organochlorine pesticides directly target the nervous system as their mechanism of toxicity [8,9,10]. Among these pesticides, OPs have received greatest attention. Other types of pesticides, including pyrethroid insecticides, herbicides, fungicides, and fumigants, have been associated with neurotoxicity [11]. Although acute poisoning disorders have been well described, there has been much disagreement on the cumulative neurotoxic effects of low-level pesticide exposure [12]. In addition to neurological symptoms, chronic low-level pesticide exposure is associated with increased prevalence of general symptoms, such as headache, dizziness, fatigue, and difficulty breathing [10,13,14,15,16,17]. In comparison, fewer investigations have been performed to assess changes in neurological signs, such as reflex, sensation, and muscle strength. Some studies suggest that vibration sensation is not affected by chronic pesticide exposure [14,18]. Whereas, others detected elevated vibration threshold sensitivity among pesticide users [19]. Furthermore, higher vibration threshold scores for the toes and increases in risk of peripheral nerve symptoms, poor coordination, abnormal deep tendon reflexes, and reduced power were detected in a rural population [20]. Of the most commonly used pesticides, exposure to OP pesticides have been associated with multiple neurological abnormalities [21].

To determine the effects of pesticide use on the health of farmers, we conducted a cross-sectional study focused on the association between neurological dysfunctions and pesticide exposure. To the best of our knowledge, this is the first large-scale field study to determine the neurological effects of pesticide exposure in China, and the findings may provide new perspectives to health policy makers.

## 2. Materials and Methods

### 2.1. Sample Design

The study was approved by the Ethics Committee of Chinese People’s Liberation Army General Hospital and all farmers sampled gave their informed, written consent. The field study took place in the provinces of Guangdong, Jiangxi, and Hebei. These provinces were selected to represent different levels of pesticide application in agricultural production in China. In Guangdong Province, where the climate is warm and humid, there are pests during all seasons and the crop system is complex. Therefore, the level of pesticide application is high. Jiangxi Province is located to the north of Guangdong, and Hebei is in Northern China.

Study participants were selected at random. Two counties from each participating province were randomly selected: Lianjiang and Xuwen of Guangdong Province; Jiujiang and Jiujiang Economic Development Zone of Jiangxi Province; Hejian and Qinghe of Hebei Province. Two villages from each county, and 20–25 households from each village were then chosen at random, generating a total of 245 study participants. Nine participants were excluded: seven participants had diabetes, one participant had cubital tunnel syndrome, and another participant underwent chemotherapy for acute lymphocytic leukemia. A total of 236 farmers participated in the study (99.6% response rate). To minimize the effect of acute pesticide exposure, the investigation took place in March 2012 before the crop season for that year.

### 2.2. Assessment of Pesticide Exposure

Exposure was determined using an interviewer-administered questionnaire, including the data of pesticide application frequency in the 3 years preceding this study (2009–2012). Based on the preliminary test, farmers in Guangdong apply pesticides most intensively. The average frequency of pesticide application in Guangdong is ~50 times over a period of 3 years. Thus, we used ≥50 times as a measure of the most intensive pesticide use. Farmers who sprayed ≥50 times during that period were classified into the high-level exposure group (Group H), and those who sprayed <50 times were classified into the low-level exposure group (Group L).

### 2.3. Data Collection

Demographic information was collected for each study participant. A detailed personal medical history was recorded, including information on the presence or a family history of neurological disorders, thyroid disease, malignancy, history of pesticide poisoning, medication use, and use of substances such as tobacco and alcohol.

A questionnaire was used to identify neuropathic symptoms, including muscle weakness, sensory disturbances, and autonomic symptoms [22]. The presence or absence of symptoms was recorded as “yes” or “no”, respectively. Neurological physical examinations were conducted to identify the presence of clinically evident signs of motor and sensory dysfunction, especially in the peripheral nervous system. Standard neurological physical examinations as described previously [23] were performed by 2 certified neurologists who had no prior knowledge of pesticide exposure for each of the participants. Evaluations included bilateral muscle strength (including bilateral intrinsic hand and foot muscles), coordination, Romberg’s sign, sensation (pinprick, vibration), deep tendon reflexes, and orthostatic hypotension if relevant symptoms presented. Pinprick and vibration perception tests were performed using a disposable safety needle and 128 Hz tuning fork, respectively. Peripheral nerve dysfunction was defined as the presence of symmetrical stocking or stocking–glove decrease of pinprick/vibration sensation, together with a reduction in ≥2 tendon reflexes [24]. Results of clinical examinations were recorded with binary indictors (normal *vs.* abnormal). The procedures and standards of the survey were well described, and both neurologists received sufficient training before embarking on the study.

### 2.4. Statistical Analysis

Statistical analysis was performed with STATA software (version 11.0). Study participants were categorized into Groups H and L based on pesticide use. Comparisons of participant characteristics were made between the two groups using Student’s *t*-tests for continuous variables and Chi-square tests for categorical variables. The odds ratios (OR) and 95% confidence intervals (CI) for neurological deficits and high exposure level were calculated using univariate and multivariate logistic regression analysis. Control variables used in regression models were age, years of education, gender, body mass index (BMI), exposure to other potentially neurotoxic substances, and dummy variables indicating the location (province) and the use of tobacco or alcohol. To evaluate the cumulative effects of low-level pesticides exposure, recent pesticide exposure was incorporated in all models. Statistical tests were two-sided, and *p*-values of <0.05 were considered statistically significant.

## 3. Results and Discussion

### 3.1. Characteristics of Farmers by Pesticide Exposure

Table 1 summarizes the characteristics of study participants by pesticide exposure. No statistical differences were found between the two groups in years of education, gender, substance use (tobacco and alcohol), and protective measures. However, farmers in Group L were older, had a higher BMI, and had greater exposure to potentially neurotoxic chemicals, including paint, gasoline, carbon monoxide, hydrogen sulfide, and sulfuric acid. Participants in Group H had more poisoning events (17.4% *vs.* 6.2%), and were likely to use pesticides in the past 30 days (51.0% *vs.* 8.6%) than Group L participants. The pesticides used in past 30 days were predominantly herbicides and acaricides. Insecticides were rarely used. Most farmers from Guangdong (87.5%), where the major crops are rice, vegetables, and orchard fruits, were exposed to a higher level of pesticides. 84% of farmers in Jiangxi Provinces applied a higher level reserved for pest control of rice, cotton, and rapeseed production. In contrast, farmers in Hebei Province use relatively fewer pesticides and mainly for wheat, maize, and cotton crops, with 27.2% farmers in Group H (Table 2).

**Table 1:** Baseline characteristics of 236 farmers according to the level of pesticide exposure.

Variables	Pesticides Application in 2009–2011		*p* Value
	Group H	Group L	
No. of farmers	155	81	
Mean age, y (SD)	50.2	53.8	0.005 **
Mean education year, (SD)	7.07	7.52	0.19
Female, %	23.9	33.3	0.12
Current smoker, %	50.3	39.5	0.11
Current drinker, %	41.3	42.0	0.92
Mean body mass index (SD)	22.8	24.4	0.001 **
Protection measure, %	14.2	12.4	0.69
Pesticide poisoning events, %	17.4	6.2	0.02 *
Recent pesticide use, %	51.0	8.6	<0.001 **
Exposure to other potentially neurotoxic substances, %	0.65	4.94	0.03 *

Note: * and ** indicate significance at 5% and 1%, respectively.

**Table 2:** Exposure levels of farmers and major crops in different regions in 2009–2011.

Province	Group H % (n)	Group L % (n)	Major Crops
Guangdong	87.5 (70)	12.5 (10)	rice, vegetables, orchards
Jiangxi	84.0 (63)	16.0 (12)	rice, cotton, rapeseed
Hebei	27.2 (22)	72.8 (59)	wheat, maize, cotton

Previous studies compared farm workers or farmers exposed to pesticides with unexposed groups [13,14,15,17,19,25] or defined indirect exposure groups [20,26]. Even unexposed individuals will encounter pesticides at some point [11], and this will underestimate the effects of pesticide exposure. Instead of recruiting healthy, unexposed controls or assigning farmers to exposed and unexposed groups, we opted to divide farmers into two groups based on their actual pesticide use. Natural variation of pesticide exposure levels was achieved by selecting three provinces with different exposure levels in Northern and Southern China. Our study design differed from other published research because our study participants were all farmers living in the same environment, which made them more comparable. Considering there are hundreds of pesticides used, with complex formulations, it was not possible to narrow down the exact quantity of each pesticide type used in the 3 years prior to this study. Therefore, we selected frequency of use to evaluate level of pesticide exposure on the basis of the following assumptions: (1) the frequency of use of each type of pesticide is similar among the three provinces evaluated; (2) farmers within each province use the same type and amount of pesticide against particular pests; and (3) the size of farmland does not vary within a region. For those reasons, the health effects of pesticides predominantly depend on the frequency of spraying and the location (province). We were able to provide an estimate of pesticide exposure level from spraying frequency and by adjusting for provincial dummy variables in the logistic regression model.

### 3.2. Neurological Symptoms

Univariate analysis of neurological physical examinations indicated that symptoms of numbness or prickling, and orthostatic dizziness or fainting were significantly associated with high-level pesticide exposure (Table 3). Other symptoms, including loss of muscle strength, unsteadiness when walking, sweating abnormalities, frequent constipation or diarrhea, and impotence had an increased prevalence. However, differences between the 2 groups were not statistically significant.

**Table 3:** Univariate analysis of neurological symptoms according to the level of pesticide exposure.

Variables	Level of Pesticide Exposure in 2009–2011 % (n)		Χ^2^ for Trend	*p* Value
	High	Low		
Had symmetrical “numbness” or “prickling” at fingers or toes?	32.3 (50)	18.5 (15)	5.03	0.03 *
Had pain or burning at any location?	20.7 (32)	24.7 (20)	0.51	0.48
Had any loss of muscle strength in your limbs?	12.9 (20)	6.2 (5)	2.54	0.11
Been unsteady when you walk?	1.9 (3)	1.2 (1)	0.16	0.69
Been sweating when it is not hot?	19.4 (30)	14.8 (12)	0.75	0.39
Been fainted or dizzy when stand up?	21.3 (33)	8.6 (7)	6.05	0.01 *
Had trouble controlling your bladder?	6.5 (10)	8.6 (7)	0.38	0.54
Had constipation or diarrhea frequently?	11.6 (18)	11.1 (9)	0.01	0.91
Had problems with erections?	6.5 (10)	6.2 (5)	0.01	0.93

Note: * indicate significance at 5%.

Logistic regression analysis showed a significantly higher OR of 2.62 (95% CI, 1.08–6.36) for numbness or prickling in Group H (Table 4). Female gender (OR 4.48, 95% CI: 1.66–12.10) and exposure to neurotoxic substances (OR 8.13, 95% CI: 1.06–62.07) also increased the risk of developing the symptoms of numbness or prickling. Although the OR for loss of muscle strength was >2 (OR 3.82, 95% CI: 0.96–15.25), it did not reach statistical significance. Pesticide exposure did not increase the risk of developing other neuropathic symptoms.

Function of the autonomic nervous system was not fully assessed in previous surveys. Pilkington *et al.* [27] found that the risk of developing sensory symptoms, but not autonomic symptoms, such as sweating, fainting, and impotence, were significantly increased in sheep dippers who handled OP concentrate. In sheep farmers and dippers, small nerve fiber function assessed by hot or cold sensation, was more prevalent than large fiber function assessed by vibration threshold or sural nerve function [10]. Similarly, the authors did not detect any statistical association between autonomic symptoms and pesticide exposure [27]. In the present study, we revealed that symmetrical numbness and prickling sensations were associated with cumulative pesticide spraying activity in a length-dependent distribution, which may indicate that small nerve fibers were affected. Muscle weakness and impairment of vibration sensitivity were caused by large fiber damage. Therefore, it is indicated that somatosensory small fibers are more likely to be affected than large fibers following pesticide use among Chinese farmers.

**Table 4:** Logistic regression models predicting neurological symptoms.

Variables	Numbness or Prickling			Loss of Muscle Strength			Urinary Incontinence			Constipation or Diarrhea		
	OR	95%CI	*p Value*	OR	95%CI	*p Value*	OR	95%CI	*p Value*	OR	95%CI	*p Value*
Group H (Yes = 1, No = 0)	2.62	1.08–6.36	0.03 *	3.82	0.96–15.25	0.06	1.22	0.31–4.84	0.77	1.51	0.47–4.88	0.49
Female (Yes = 1, No = 0)	4.48	1.66–12.10	0.003 **	3.11	0.79–12.32	0.11	3.25	0.63–16.66	0.16	1.05	0.26–4.30	0.95
Education year	1.09	0.98–1.22	0.11	1.03	0.88–1.21	0.69	0.95	0.80–1.13	0.55	1.04	0.89–1.21	0.62
Age	1.01	0.98–1.04	0.68	1.01	0.96–1.06	0.81	0.98	0.93–1.03	0.47	1.01	0.97–1.06	0.63
BMI	0.94	0.85–1.05	0.28	0.92	0.79–1.07	0.31	0.90	0.76–1.06	0.22	0.89	0.76–1.03	0.11
Current smoker (Yes = 1, No = 0)	2.09	0.93–4.66	0.07	1.68	0.51– 5.57	0.39	2.02	0.47–8.63	0.34	2.98	0.99–9.01	0.05 *
Current drinker (Yes = 1, No = 0)	0.66	0.33–1.32	0.24	0.36	0.12–1.08	0.07	0.63	0.18–2.23	0.47	0.22	0.08–0.64	0.005 **
Other neurotoxic substances exposure	8.13	1.06–62.07	0.04 *	17.04	1.95–149.13	0.01 **	13.24	1.49–117.51	0.02 *	7.81	1.05–58.22	0.05 **
Guangdong	1.30	0.48–3.53	0.61	0.87	0.20–3.80	0.85	0.76	0.16–3.73	0.74	0.59	0.16–2.21	0.43
Jiangxi	0.78	0.29–2.13	0.63	0.74	0.17–3.16	0.69	0.26	0.05–1.51	0.13	0.28	0.07–1.23	0.09

Note: * and ** indicate significance at 5% and 1%, respectively.

### 3.3. Neurological Signs

Based on univariate analysis of the data, farmers in Group H had increased impairment of coordination and sense of vibration (Table 5). Logistic regression analysis showed that impairment of vibration sensation had an OR > 2 (OR 2.49, 95% CI 0.83–7.47), however, this did not reach statistical significance (Table 6). In this model, female gender and smoking were positively associated with a reduction in vibration sensitivity. Reductions in pinprick sensitivity and deep tendon reflexes were not significantly associated with pesticide exposure.

**Table 5:** Univariate analysis of neurological signs according to the level of pesticide exposure.

Variables	Level of Pesticide Exposure in 2009–2011 % (n)		Χ^2^ for Trend	*p* Value
	High	Low		
Muscle strength	2.6 (4)	3.7 (3)	0.23	0.63
Coordination	9.7 (15)	2.5 (2)	4.14	0.04 *
Romberg sign	1.3 (2)	1.2 (1)	0.001	0.97
Pinprick sensibility	14.2 (22)	11.1 (9)	0.44	0.51
Vibration sensibility	25.2 (39)	7.4 (6)	10.87	0.001 *
Deep tendon reflexes	16.8 (26)	11.1 (9)	1.35	0.25
Orthostatic hypotension	0.7 (1)	0 (0)	0.52	0.47

Note: * indicate significance at 5%.

**Table 6:** Logistic regression models predicting neurological signs.

Variables	Pinprick Sensibility			Vibration Sensibility			Deep Tendon Reflexes		
	OR	95%CI	*p* Value	OR	95%CI	*p* Value	OR	95%CI	*p* Value
Group H (Yes = 1, No = 0)	0.97	0.34–2.75	0.96	2.49	0.83–7.47	0.10	1.38	0.48–3.99	0.55
Female (Yes = 1, No = 0)	5.29	1.41–19.82	0.01 **	3.81	1.10–13.14	0.03 *	1.54	0.42–5.66	0.52
Education year	1.26	1.07–1.48	0.01 **	1.08	0.94–1.24	0.26	1.05	0.91–1.22	0.51
Age	0.98	0.94–1.03	0.44	0.99	0.95–1.03	0.70	1.05	1.00–1.09	0.04 *
BMI	1.14	0.99–1.30	0.06	1.00	0.88–1.14	0.97	1.07	0.94–1.22	0.31
Current smoker (Yes = 1, No = 0)	2.52	0.87–7.24	0.09	3.36	1.21–9.35	0.02 *	1.02	0.98–1.06	0.14
Current drinker (Yes = 1, No = 0)	1.16	0.47–2.85	0.75	0.59	0.26–1.32	0.20	1.02	0.99–1.05	0.13
Other neurotoxic substances exposure	3.22	0.29–35.89	0.34	6.09	0.47–78.33	0.17	7.93	0.97–65.13	0.05 *
Guangdong	2.13	0.50–9.05	0.31	3.35	0.70–15.96	0.13	1.41	0.35–5.64	0.63
Jiangxi	9.50	2.37–38.12	0.001 **	12.70	2.83–57.08	0.001 **	5.32	1.47–19.26	0.01 **

Note: * and ** indicate significance at 5% and 1%, respectively.

### 3.4. Neurologic Effects of Cumulative Low-level Exposure to Pesticides

After adjusting for recent exposure to pesticides, logistic regression analysis revealed a statistically significant association between symptoms of numbness or prickling and higher pesticide exposure (OR 2.55, 95% CI: 1.04–6.25). Loss of muscle strength and decreased vibration sensitivity were associated with high pesticide exposure if the level of significance was set at <0.1 (Table 7). Recent use of pesticide was not associated with any neurological symptoms or signs, or pesticide poisoning events (Tables S1 and S2).

The findings of the present study indicate that the most prominent neurological dysfunction associated with cumulative pesticide exposure was the sensation of numbness or prickling. There may also be an association between pesticides exposure and loss of muscle strength or decreased vibration sensitivity, however, these occurred with lower probability. Of the confounding factors, female gender and smoking were identified as risk factors for developing peripheral neuropathy. Previous studies have reported an association between cigarette smoking and neuropathy [24,28]. However, little is known as to why females are more susceptible to peripheral neuropathy. This finding may be explained by difference in the division of labor. For instance, women perform additional tasks (e.g., knitting and washing clothes) by hand.

Current knowledge of the neurologic effects associated with pesticide exposure can be categorized into acute and chronic effects of high-level exposure, and acute and cumulative effects of low-level exposure. In our study, we evaluated the cumulative effects of low-level pesticide exposure. Firstly, it was conducted before the crop season started. Although a proportion of farmers had already begun spraying pesticides, the health effects of acute pesticides exposure were not significant (Table 7). Secondly, all study participants with a history of pesticide poisoning fully recovered, and no events were associated with any neurological outcomes (Tables S1 and S2).

**Table 7:** Logistic regression models after adjusting for recent pesticide use.

Variables	Group H			Recent Pesticide Use		
	OR	95%CI	*p* Value	OR	95%CI	*p* Value
Numbness or prickling	2.55	1.04–6.25	0.04 *	1.24	0.52–2.96	0.63
Loss of muscle strength	3.54	0.86–14.64	0.08	2.19	0.63–7.67	0.22
Urinary incontinence	1.21	0.30–4.85	0.79	1.20	0.21–6.88	0.84
Constipation or diarrhea	1.31	0.38–4.46	0.67	3.57	0.73–17.44	0.12
Pinprick sensibility	1.05	0.37–2.99	0.93	0.55	0.18–1.68	0.93
Vibration sensibility	2.53	0.83–7.69	0.10	0.92	0.37–2.32	0.86
Deep tendon reflexes	1.48	0.51–4.31	0.47	0.65	0.23–1.83	0.41

Note: * indicate significance at 5%. Other control variables, including female, education year, age, BMI, current smoker, current drinker, neurotoxic substances exposure and province variables were included, but not reported.

### 3.5. Study Limitations

In the present study, the total number of times pesticides were sprayed in the 3 years preceding the study was used to estimate relative exposure levels. Relying on participants to recall this information was a likely source of measurement error. A prospective cohort study with long-term follow-up would provide a more accurate assessment of exposure. Furthermore, information regarding previous history of pesticide poisoning was collected during face-to-face interviews rather than from medical records. This may have presented further recall bias. The clinical neurological findings collected were subjective and qualitative. Objective and quantitative measures, such as studies of nerve conduction, quantitative sensory testing, and other neurophysiologic tests would enable the detection of subclinical effects of pesticide use. It should also be noted that the neurological effects detected in our study resulted from the use of multiple pesticides. Further investigation is necessary to differentiate the neurological effects of specific pesticides.

## 4. Conclusions

Neurological symptoms of numbness and sensations of prickling were related to pesticide exposure among farmers in China. These results indicate that overuse has adverse impacts on farmers’ health. Informing farmers of these findings might help them make a conscious effort to reduce the extent of pesticide use.

## Supplementary Files

Supplementary File 1 Supplementary Information (PDF, 128 KB)
