# Supplementary material for: Neurological Effects of Pesticide Use among Farmers in China
Source: Int J Environ Res Public Health. 2014 Apr 14;11(4):3995–4006. doi: 10.3390/ijerph110403995 (PMC4025041; doi:10.3390/ijerph110403995)
Supplement: Supplementary File 1 — Supplementary Information (PDF, 128 KB) [file ijerph-11-03995-s001.pdf]

Neurological Effects of Pesticide Use among Farmers in China

Table S1. Logistic regression models predicting neurological symptoms adjusting for pesticides poisoning history.

Variables	Numbness or prickling	Muscle Strength	Urinary Incontinence	Constipation or Diarrhea
Group H	0.92 *	1.12	0.17	0.30
(Yes = 1, No = 0)	(0.46)	(0.71)	(0.72)	(0.61)
Pesticides poisoning history	0.42	1.28 *	0.15	0.66
(Yes = 1, No = 0)	(0.44)	(0.55)	(0.74)	(0.57)
Other neurotoxic agents exposure	2.12 *	2.97 **	2.59 *	2.12 *
(Yes = 1, No = 0)	(1.03)	(1.09)	(1.11)	(1.03)
Female	1.48 **	1.08	1.16	−0.01
(Yes = 1, No = 0)	(0.51)	(0.70)	(0.84)	(0.72)
Education year	0.09	0.05	−0.05	0.05
	(0.06)	(0.08)	(0.09)	(0.08)
Age	0.01	0.01	−0.02	0.01
	(0.02)	(0.03)	(0.03)	(0.02)
BMI	−0.05	−0.07	−0.11	−0.12
	(0.05)	(0.08)	(0.09)	(0.07)
Current smoker	0.73	0.50	0.70	1.09
(Yes = 1, No = 0)	(0.41)	(0.62)	(0.74)	(0.57)
Current drinker	−0.40	−0.98	−0.46	−1.52 **
(Yes = 1, No = 0)	(0.36)	(0.57)	(0.65)	(0.55)
Guangdong	0.22	−0.34	−0.28	−0.61
	(0.51)	(0.77)	(0.81)	(0.68)
Jiangxi	−0.21	−0.10	−1.30	−1.18
	(0.52)	(0.75)	(0.90)	(0.75)
Constant	−2.17	−2.51	0.85	−0.21
	(1.79)	(2.64)	(2.83)	(2.48)
Observations	236	236	236	236

Notes: * and ** indicate the statistically significant at 5% and 1%, respectively. Robust standard errors are in parentheses.

Table S2. Logistic regression models predicting neurological signs adjusting for pesticides poisoning history.

Variables	Pin Sensibility	Vibration Sensibility	Deep Tendon Reflexes
Group H	−0.04	0.83	0.28
(Yes = 1, No = 0)	(0.53)	(0.57)	(0.55)
Pesticides poisoning history	0.13	0.74	0.68
(Yes = 1, No = 0)	(0.62)	(0.51)	(0.55)
Other neurotoxic agents	1.19	1.92	2.15 *
exposure (Yes = 1, No = 0)	(1.23)	(1.30)	(1.08)
Female	1.66 *	1.33 *	0.44
(Yes = 1, No = 0)	(0.67)	(0.63)	(0.66)
Education year	0.23 **	0.09	0.06
	(0.08)	(0.07)	(0.07)
Age	−0.02	−0.01	0.05 *
	(0.02)	(0.02)	(0.02)
BMI	0.13	0.005	0.07
	(0.07)	(0.07)	(0.07)
Current smoker	0.92	1.22 *	0.73
(Yes = 1, No = 0)	(0.54)	(0.53)	(0.52)
Current drinker	0.15	−0.49	0.67
(Yes = 1, No = 0)	(0.46)	(0.42)	(0.43)
Guangdong	0.74	1.12	0.29
	(0.74)	(0.80)	(0.72)
Jiangxi	2.27 **	2.66 **	1.73 **
	(0.71)	(0.78)	(0.67)
Constant	−8.02 **	−4.96 *	−8.11 **
	(2.66)	(2.43)	(2.54)
Observations	236	236	236

Notes: * and ** indicate the statistically significant at 5% and 1%, respectively. Robust standard errors are in parentheses.

© 2014 by the authors; licensee MDPI, Basel, Switzerland. This article is an open access article distributed under the terms and conditions of the Creative Commons Attribution license (http://creativecommons.org/licenses/by/3.0/).
